# Molecular investigations of viral meningitis among HIV-infected adults in Accra, Ghana

**DOI:** 10.1186/s13104-018-3720-z

**Published:** 2018-08-28

**Authors:** Emmanuel Frimpong Adjei, Theophilus Korku Adiku, Gifty Mawuli, Joseph Humphrey Kofi Bonney

**Affiliations:** 1 Department of Medical Microbiology, School of Biomedical and Allied Health Sciences, College of Health Sciences, University of Ghana, Accra, Ghana; 2 Noguchi Memorial Institute for Medical Research, College of Health Sciences, University of Ghana, Accra, Ghana

**Keywords:** Human immunodeficiency virus, Meningitis, Cerebrospinal fluid, Viral aetiology

## Abstract

**Objective:**

Meningitis is one of the leading causes of death among patients living with the human immunodeficiency virus (HIV) in sub-Saharan Africa. Based on clinical presentations alone, the different types of meningitis may not be distinguished from each other, consequently accurate laboratory diagnosis is extremely essential. Viruses such as Enteroviruses (EV), Mumps virus (MuV) and Herpes Simplex Virus-1 (HSV-1) are implicated in cases of meningitis. We sought to detect and characterize viral aetiologies of meningitis among HIV-infected adults with the use of molecular tools.

**Results:**

As a subset of a main research work, cerebrospinal fluid specimens were collected from a cross-section of HIV patients at the Fevers Unit of the Korle Bu Teaching Hospital with clinical features suggestive of meningitis but without laboratory confirmation. Laboratory investigations were performed with the use of the real time polymerase chain reaction for pan EV, MuV and HSV-1. None of the viruses investigated in this study was found to be positive for meningitis. However, lymphocytic pleocytosis, normal glucose and elevated protein levels were observed in some of the study participants.

## Introduction

Viral meningitis is a central nervous system (CNS) related condition which is self-limiting but associated with high rate of morbidity among immunocompromised populations [1, 2]. In general, meningitis as a condition has been associated with several aetiologies such as bacteria, parasites, fungi or viruses [3–5]. Different viral agents have been implicated as aetiologies of viral meningitis with the prevalence recorded in several studies [6–9]. Enteroviruses, particularly echovirus 22 and 23 (also known as Human parechoviruses type 1 and 2) have been documented as the second cause of viral meningitis in young children [10, 11]. Additionally, Herpes Simplex Virus-1 (HSV-1) and Mumps virus (MuV) are also known to cause meningitis [12–15].

The possibility of a viral aetiology of meningitis usually arises once bacterial and fungal stains or cultures of cerebrospinal fluid (CSF) are negative. Amidst the myriad of illnesses presented by a patient with meningitis, it is documented that viral meningitis is often associated with lymphocytic pleocytosis, normal glucose and elevated protein [3, 16, 17]. Based on clinical presentations alone, the different causes of meningitis may not be clearly known which makes the proper management and treatment of the patients difficult. Accurate diagnosis is therefore essential and will help reduce the indifferent use of antibiotics, hospital visits and medical bills in such a resource limited setting.

Nucleic acid amplification techniques provide a rapid and specific aetiological diagnosis for cases of meningitis. Other diagnostic assays such as culture and immunoassays are less sensitive and take a longer time for results to be attained. Further to that, virus isolation attempts by culture methods have not been successful for all the known meningitis-causing viruses [18].

Predominantly, more studies in Ghana have documented the contribution of other microbial agents including bacteria and parasites as aetiologies of meningitis [19–21, 25], with limited work on viral agents implicated in neurologic infections. Cases of clinically suspected meningitis are recorded at the FU of KBTH but confirmation and further identification of the aetiological agents are rarely done. In a broader study [25], we investigated and detected varied microbial pathogens in 51 out of 84 HIV-infected adults with provisional diagnosis of meningitis in the following distribution: Epstein–Barr virus 28.6%, *Toxoplasmosis* 25.0 and 2.4% each for Cytomegalovirus and *Cryptococcus*. This added-on research sought to use molecular diagnostic tools to determine the occurrence of other known viral aetiologies documented to cause and exacerbate meningitis in especially immunocompromised patients.

## Main text

### Methods

This study is an addition to a main research work in which other aetiological agents that could cause meningitis were investigated. This current study takes into consideration three viruses (EV, MuV and HSV-1) which are also known to cause meningitis.

#### Study area and subjects

This study was conducted at the Fevers’ Unit (FU) of the Korle Bu Teaching Hospital (KBTH) in Korle Bu, Accra from August 2014 to January 2016. Korle Bu Teaching Hospital is a referral hospital with a 2000 patient bed capacity and 17 clinical and diagnostic departments and the largest tertiary health facility in Accra which is the capital city of Ghana. In all, eighty (80) patients who had been tested and confirmed to be HIV positive and with clinical symptoms consistent with meningitis were recruited for this study. All patients were adults (≥ 18 years). As meningitis may present with diverse clinical symptoms such as headache, fever, stiff neck, neurologic symptoms, abnormal behaviour, seizure, nausea, tachycardia, photophobia and others, it was expedient to have a clear definition for cases. Trained health staff screened and enrolled patients who met the standard case definition of meningeal symptoms of stiff-neck, fever and headache as well as cerebrospinal fluid pleocytosis, with no laboratory evidence of bacterial or fungal organisms. .

#### Sample collection

The archived clinical specimens collected from our previous study [25] were used for this added-on research work. For each patient, the CSF specimens were collected were kept in two separate bottles (sterilized Bijou sample container and a Shutterstock Sodium fluoride containing bottle; Mersk KGaA, Darmstadt, Germany). The CSF specimens in fluoride bottles were used to ascertain CSF biochemical parameters at the Central Laboratory of KBTH and that in the Bijou bottle was used to perform initial microbiological analyses such as CSF cell count and differential at the Medical Microbiology Department research laboratory of University of Ghana. Residual specimens were then cryopreserved in a − 20°C freezer until they were transported in a cool box with ice packs to the Noguchi Memorial Institute for Medical Research (NMIMR) for further processing.

#### Biochemical data

Cerebrospinal fluid biochemical parameters that were measured included White cell counts and differential, total protein levels, glucose levels and globulin assay (Table 1) with the use of an automated hematological analyzer (Sysmex XN-1500, Sysmex, Europe). Glucose level in the CSF was measured using the Glucose oxidase method [26]. A turbidimetric method [27] was used to ascertain the protein levels of the CSF samples using trichloroacetic acid.

**Table 1:** Laboratory findings of CSF samples based on cellular and biochemical characteristics

Variables	Frequency	Percentage
WBC differential [(cells/mm^3^); (normal range)]		
Lymphocytes	30	75.00
PMNs	7	17.50
PMNs/lymph	3	7.5
CD4 count (cell/mm^3^)		
Mean/SD	*1383.96* ± *3285.23*	
Range[(min–max); (normal range)]	(*1*–*979*)	
< 200 (500–1500)	53	66.25
> 200 (500–1500)	14	17.50
CSF-glucose (mmol/l)		
Mean/SD	*2.9025* ± *1.22*	
Range [(min–max); (normal range)]	(*1.00*–*8.80*)	
< 2.5 (2.5–4.4)	21	26.25
≥ 2.5 (2.5–4.4)	59	73.75
CSF-protein (g/l)		
Mean/SD	*1.1498* ± *1.56*	
Range [(min–max); (normal range)]	(*0.03*–*9.86*)	
≤ 0.45 (0.15–0.45)	26	32.50
> 0.45 (0.15–0.45)	54	67.50
CSF globulin (normal range − 3 to 12% of CSF total protein)		
Positive	48	60
Negative	29	36

0–5 cells/µl (< 2 polymorphonucleocytes [PMN]) normal cell counts do not rule out meningitis or any other pathology

Mean/SD values and the normal ranges for the variables considered are in italics

#### PCR assays: nucleic acid extraction, quantification and amplification

The extraction and purification of nucleic acid was performed using QIAamp Viral Mini Extraction Kit (Qiagen, Hilden, Germany). Nucleic acid was extracted from 140 μl of CSF samples from enrolled participants. The concentration and purity of the nucleic acid was measured using the NanoDrop 2000c (Thermo Scientific, Massachusetts, USA).

Realtime reverse transcription-PCR (rRT-PCR) was performed for EV and Muv with the reagent AgPath-ID™ One Step RT-PCR Kit (Applied Biosystems, California, USA). Samples were tested in a 25 μl reactions mixture including 5 μl nucleic acid as template, 12.5 μl of 2× RT-PCR Buffer, 1 μl of reverse transcriptase mix, 0.5 μl of each of the forward and reverse primers and probe sets. Nuclease free water (5 µl) was added to make up the mixture to 25 μl. The oligo sequences of primers and probes sets used in this study are detailed in Table 2.

**Table 2:** PCR assays used in the study

Virus	Primers oligosequences (5′–3′)	Target region	References
Enterovirus	5′-CCCTGAATGCGGCTAATCC-3′, reverse primer, 5′-ATTGTCACCATAAGCAGCCA-3′ and a probe, 5′-AACCGACTACTTTGGGTGTCCGTGTTTC-3′	5′ UTR	[22]
Mumps	Forward primer (SH61F) 5′-GTGACCCTGCCGTTGCA-3′, reverse primer (SH147R), 5′-GTTATGATCAGAGAGAGAAGAATTAGCAATAG-3′ and probe (SH79P2) 5′-TATGCCGGCGATCCAACCTCCCTTATA-3′	(SH) gene	[23]
HSV-1	Forward primer (HSV1UP), 5′-CGGCCGTGTGACACTATCG-3′, reverse primer (HSV1DP), 5′-CTCGTAAAATGGCCCCTCC-3′ and probe (HSV1P), 5′-CCATACCGACCACACCGACGAACC-3′	(gD) gene	[24]

Amplification was performed using the Applied Biosystems^®^ 7300 Real time PCR instrument (Life Technologies, California, USA). Cycling conditions for all primer and probe sets consisted of a reverse transcriptase step at 45 °C for 45 min, followed by a *Taq* polymerase activation step at 95 °C for 10 min and then 40 cycles at 95 °C for 15 s (denaturing) and 60 °C for 1 min (annealing and extension step). Data were collected at the 60 °C for 1 min (annealing and extension) step.

Real time PCR was performed for HSV-1 using AmpliTaq Gold^®^ PCR Master Mix (Roche, California, USA). Samples were tested in a 25 μl reaction mix with 5 μl nucleic acid, 11 μl Universal PCR Master Mix 2×, 2.5 μl of each specific primer and probe set and 1.5 μl nuclease free water.

Amplification was performed in same machine with slight modifications including 95 °C for 10 min then 40 cycles at 92 °C for 15 s (denaturing) and 60 °C for 1 min (annealing and extension step).

### Results and discussion

#### Quantification of nucleic acids

The integrity of the nucleic acid extracted for the 80 CSF samples were of high quality with 77 having a high yield within the range of 53.2 to 101.4 ng/μl suitable for molecular studies when measured with the Nano Drop 2000c spectrophotometer. Three had the low yield being 20.5, 18.1 and 14.3 ng/μl.

#### Real time PCR

In this study, the test run for each PCR performed involved test samples in duplicates with positive and negative controls to validate each test run. Until the positive and the negative control indicators are correctly shown after each run, the test cannot be considered valid. A repeat run was carried out in instances where none of the control indicators showed after the test run. All the PCR test runs for this work were validated.

**Fig. 1:**
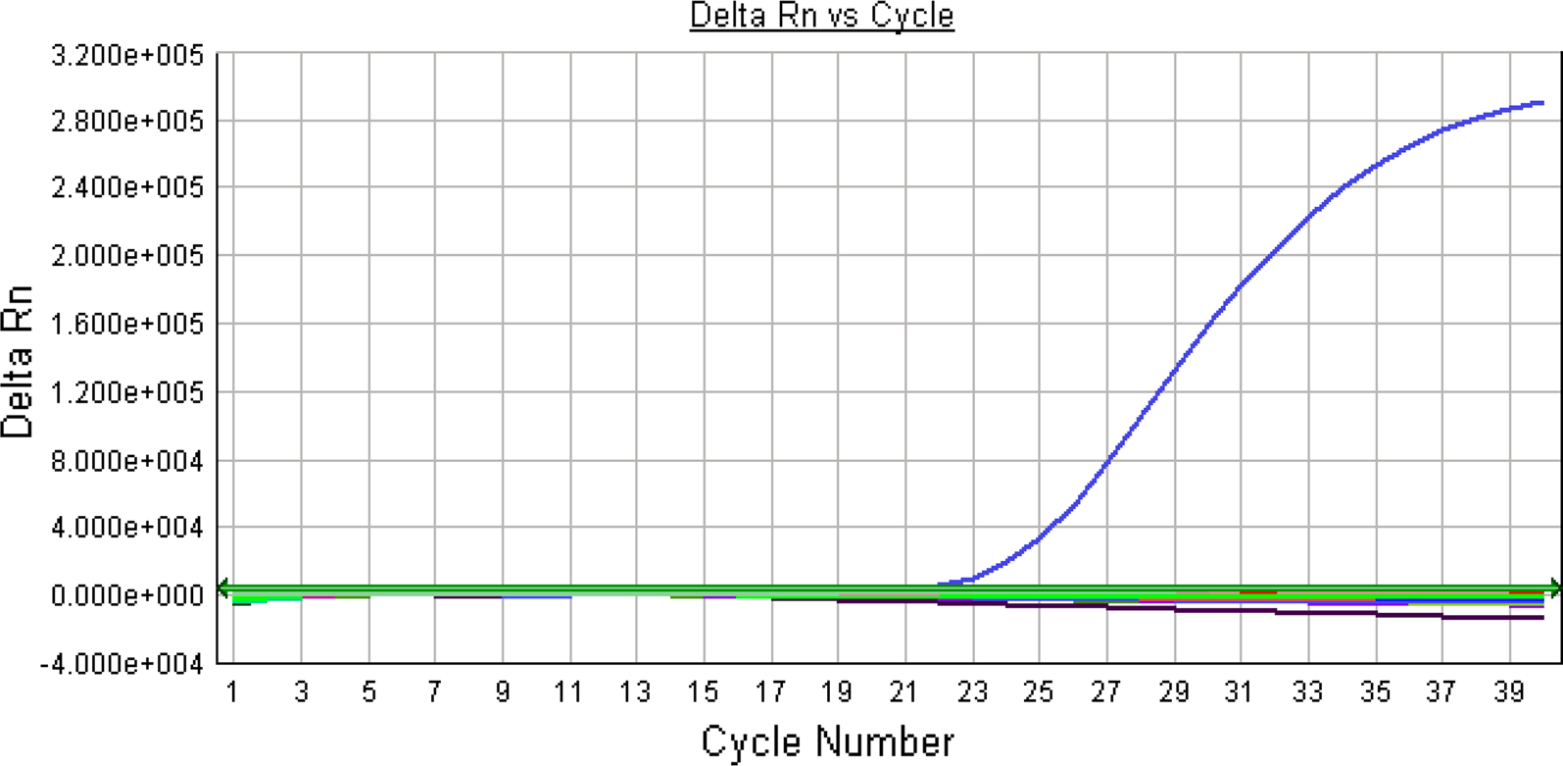
Figure representing the amplification curve of the Real-time PCR runs for the samples and controls. The horizontal line with arrows represents the threshold. The curve that crossed the threshold represents the positive control. The samples and negative controls are below the threshold line (irregular lines) which represent a negative result. The amplification of the positive control depicts a successful PCR run

None of the 80 suspected cases of meningitis investigated by PCR tested positive for EV, MuV and HSV-1 as no viral nucleic acid was detected. Figure 1 represents a PCR amplification plot of a test run with a detectable amplification for the positive control whilst the negative control and the samples included in the run were undetectable hence no amplification.

#### Conclusion

None of the viruses (EV, MuV, HSV-1) investigated in this study was detected in the suspected cases of meningitis. However, lymphocytic pleocytosis, normal glucose and elevated protein levels observed in majority of study participants.

## Limitations

Our study could have been better but for some limitations. A larger and more representative sample size would have given a better information on the prevalence of the three viruses and interpretation of our results. Besides, the clinical and laboratory data for some of the patients were incomplete. Another noticeable limitation was the exclusion of other documented viral aetiologies for meningitis and a serological assay which would have provided useful information on exposure levels of the patients to the viral agents.

## Abbreviations

HIV: human immunodeficiency virus
EV: Enteroviruses
MuV: Mumps virus
HSV: Herpes Simplex Virus
CSF: cerebrospinal fluid
CNS: central nervous system
KBTH: Korle Bu Teaching Hospital
FU: Fevers Unit
PCR: polymerase chain reaction
